# Assessing the feasibility of machine learning for ancient DNA age prediction: Limitations and insights

**DOI:** 10.1093/biomethods/bpag028

**Published:** 2026-06-04

**Authors:** Maksim Kazanskii, Maria Golikova, Artem Kasianov, Anna Ivanova, Alibek Suraganov, Leonid Uroshlev

**Affiliations:** Independent Researcher; Independent Researcher; Evolutionary Approaches to Human Genetic and Cultural Diversity, BIOPOLIS/CIBIO, Campus de Vairão, Universidade do Porto, 4485-661, Porto, Portugal; Independent Researcher; Independent Researcher; Laboratory of Strucural Biology, L.A. Orbeli Institute of Physiology NAS RA, Orbeli Bros. str. 0028, Yerevan, Armenia

**Keywords:** ancient DNA, DNA damage, machine learning, age prediction, Paleogenomics, DNA degradation, batch effects

## Abstract

We investigated the possibility of estimating the age of ancient biological samples directly from their DNA damage profiles using supervised machine learning. Traditional dating methods such as radiocarbon dating or dendrochronology rely on either isotope composition or material context, while our approach exploits intrinsic molecular degradation signatures. Using damage statistics obtained from ancient DNA sequencing data, we trained several regression models to predict sample age over a temporal range of up to 10 000 years. Despite initial correlations between specific damage features and age, cross-validation and external testing revealed no statistically significant predictive signal beyond mean-based baselines. These findings indicate that, in the current formulation, DNA damage information alone is insufficient for reliable age estimation. However, this negative result empirically validates the extent to which environmental and biochemical factors dominate damage variation, effectively masking the chronological signal, thereby limiting the predictive power of current supervised learning models. We suggest that integrating contextual data, expanding labeled datasets, and incorporating physical models of DNA decay may improve future attempts. Our study thus contributes to a transparent assessment of the limitations and prospects of DNA-based fossil dating.

## Introduction

Since its emergence, ancient DNA (aDNA) research has transformed archaeology and comparative anthropology. Modern sequencing techniques now make it possible to recover and analyze DNA from samples over one million years old, with the current record reaching approximately two million years for environmental DNA and about 1.3 million years for individual organisms. These advances allow researchers to identify the origins of ancient remains and compare them both with one another and with contemporary populations. aDNA studies have even led to the discovery of previously unknown human species, such as the Denisovans [1].

Despite this progress, several challenges in aDNA data analysis remain unresolved. One of the most prominent is the ability to accurately estimate the age of a biological sample solely from its DNA sequence.

Under normal physiological conditions, random DNA damage is continuously corrected by cellular repair machinery. However, after death, these repair processes cease, initiating the first phase of DNA degradation. Intracellular nucleases are released as cellular structures break down. Once liberated, these enzymes gain access to the genome and begin cleaving long DNA molecules into shorter fragments. This process progressively reduces the genome into increasingly smaller DNA fragments [2].

Once the initial hydrolytic breakdown of DNA subsides, the molecule begins to accumulate mutations, including those driven by environmental factors such as ultraviolet radiation. Two prominent chemical processes occur during this stage: depurination and cytosine deamination.

In depurination, the N-glycosidic bond linking a purine base (adenine or guanine) to the sugar–phosphate backbone is cleaved, resulting in loss of the base and potentially causing a base skip during sequencing. In cytosine deamination, the amino group is removed from cytosine, converting it into uracil. Because standard high-throughput DNA sequencing does not faithfully read uracil residues, this modification appears in sequencing data as a cytosine-to-thymine substitution [3].

In addition to these modifications, DNA can undergo further damage related to exposure to reactive oxygen species or the external environment. The older the sample, the more damage it accumulates. Therefore, using features such as DNA molecule length, number of substitutions, and mutations, it is possible to construct a model for estimating the age of a sample. Although there is currently no single model of DNA degradation over time [4], environmental factors greatly influence DNA preservation [5]. However, there is evidence of a correlation between features such as DNA fragmentation and sample age.

However, developing a model for age estimation presents several challenges. One major source of complexity is the influence of environmental conditions. Optimal preservation requires low temperatures, dry conditions, minimal exposure to ultraviolet radiation, and limited oxygen availability. In contrast, DNA degradation accelerates in environments characterized by high or fluctuating temperatures, elevated humidity or frequent precipitation, direct sunlight, acidic or highly alkaline soils, and intense microbial activity [6].

It should also be noted that only a small number of studies have focused on investigating the dynamics of nuclear DNA decay. Much more often, authors focus on mitochondrial or bacterial DNA. The rate of nuclear DNA decay is twice that of mitochondrial DNA decay, which is why most authors prefer to work with mitochondria.

In this work, we investigate whether the chronological age of ancient biological samples can be inferred directly from their DNA damage patterns using supervised machine learning. Motivated by known associations between post-mortem molecular decay processes and sample age, we extract features such as fragment length distributions and characteristic substitution patterns from ancient DNA sequencing data and evaluate multiple regression approaches. Our goal is to assess the feasibility and limitations of DNA-based temporal prediction and determine whether damage signatures contain a stable signal that can serve as a proxy for sample age independent of contextual or isotopic information.

## Materials and methods

### Data

The age prediction for ancient DNA samples was formulated as a regression task. For model training, we used output tables generated by DamageProfiler [7], which report the frequency of each mutation type. DamageProfiler is a tool designed to characterize nucleotide misincorporation patterns in ancient DNA. It provides detailed profiles of post-mortem DNA damage, identifying and quantifying substitution types that are characteristic of ancient samples. In particular, it detects deamination-driven changes such as C→T and G→A transitions, which typically occur near the fragment ends. The transition mutation types considered in this study are as follows:

- TOTAL: Total number of nucleotides at the specified positions.
- A, C, G, T: Counts of individual nucleotides observed at the specified positions.
-
G→A , C→T, A→G, T→C, A→C, A→T, C→G, C→A, T→G, T→A, G→C, G→T**:** Single-base substitutions representing transitions or transversions from one nucleotide to another.
-
A→− , T→−, C→−, G→−**:** Deletions where a nucleotide is lost from the sequence.
-
→A , →T, →C, →G**:** Insertions where a nucleotide is added to the sequence.
-
→S : Soft-clipped bases—nucleotides located at the ends of reads that do not align to the reference genome.

**Figure 1:**
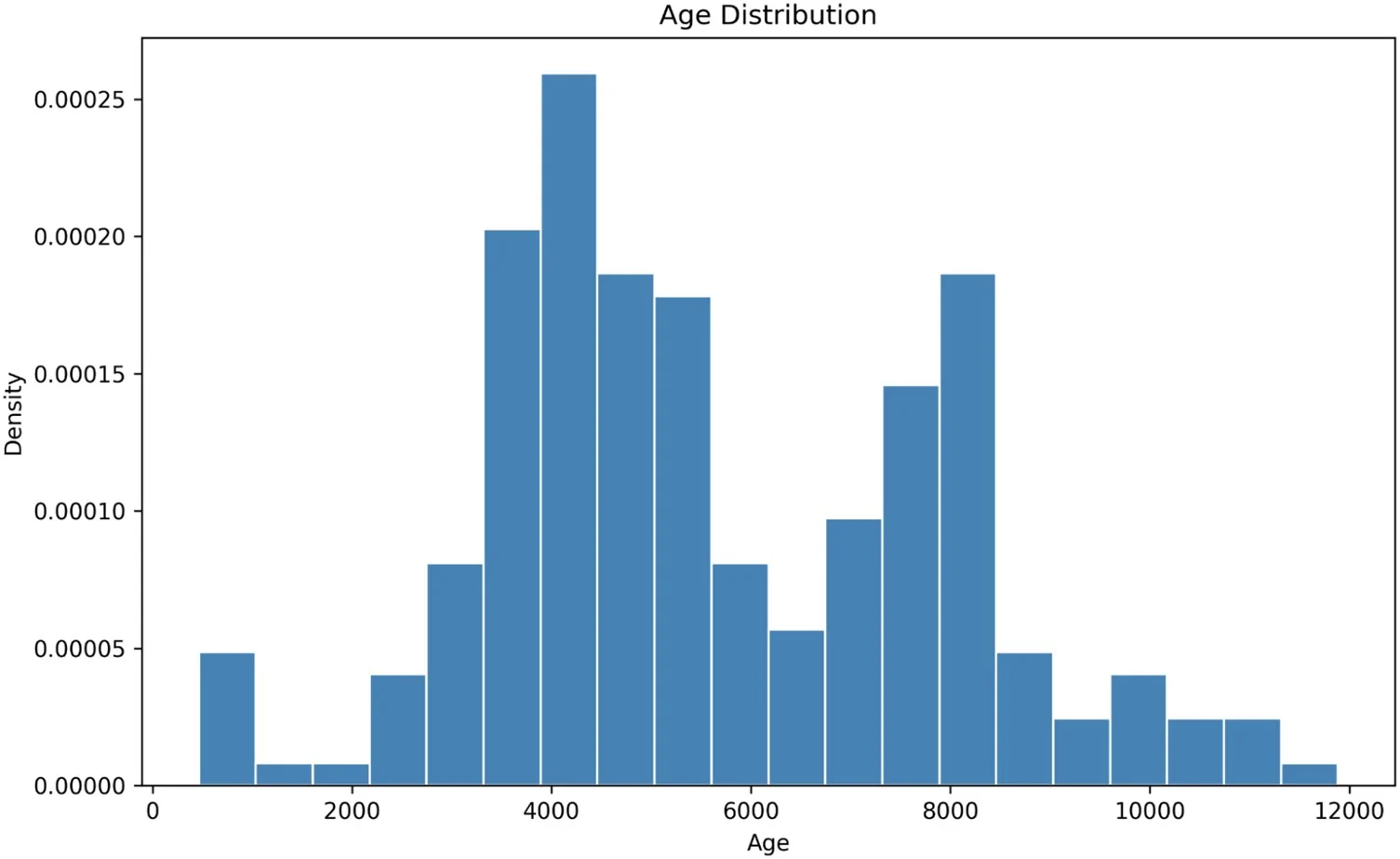
The distribution of samples in Harvard dataset by ages.

As the dataset, we used the Allen Ancient Genome Diversity Project [8] (hereafter referred to as the Harvard dataset), which contains 216 BAM files. This large-scale ancient DNA sequencing initiative aims to explore genetic diversity, identify population-specific SNPs, and reconstruct human migration patterns. The sequences in this dataset have a median coverage of approximately 5×, and the sample ages range from roughly 500 to nearly 12 000 years. The distribution of the ages of the samples is presented in Fig. 1. All reads were generated on Illumina platforms, with a mean read length of 60 bp, followed by basecalling and alignment to the hs37d5 reference genome. The hs37d5 (hg19) reference genome was chosen because it remains widely used in ancient DNA studies, ensuring compatibility with legacy datasets and tools. GRCh38, while more recent, introduces alternate haplotypes and coordinate changes that complicate alignment consistency and batch-effect control. Most samples underwent UDG treatment (uracil-DNA glycosylase) to remove uracils and improve sequencing accuracy. Additionally, a standardized bioinformatic pipeline was applied across all samples, including adapter trimming, read merging, alignment to the human reference genome, and duplicate removal. Finally, DNA damage patterns were quantified using DamageProfiler to provide the feature set for the machine learning models. This resulted in a highly uniform and consistent dataset that is well suited for model training.

In addition to the Harvard dataset, we collected an independent set of publicly available ancient genomes from multiple published studies spanning Europe, Asia, Africa, and the Americas (listed below). These samples were downloaded from the European Nucleotide Archive (ENA) and the NCBI Sequence Read Archive (SRA), and they include individuals ranging from approximately 350 to approximately 10 850 years in age. We used this additional dataset as an external test. The detailed list of samples, including extracted ages, study names, and corresponding references, is provided in Appendix 4.

We performed clustering on the DamageProfiler output and visualized the data in a two-component space for the combined dataset (Harvard dataset samples and the external test dataset).

**Figure 2:**
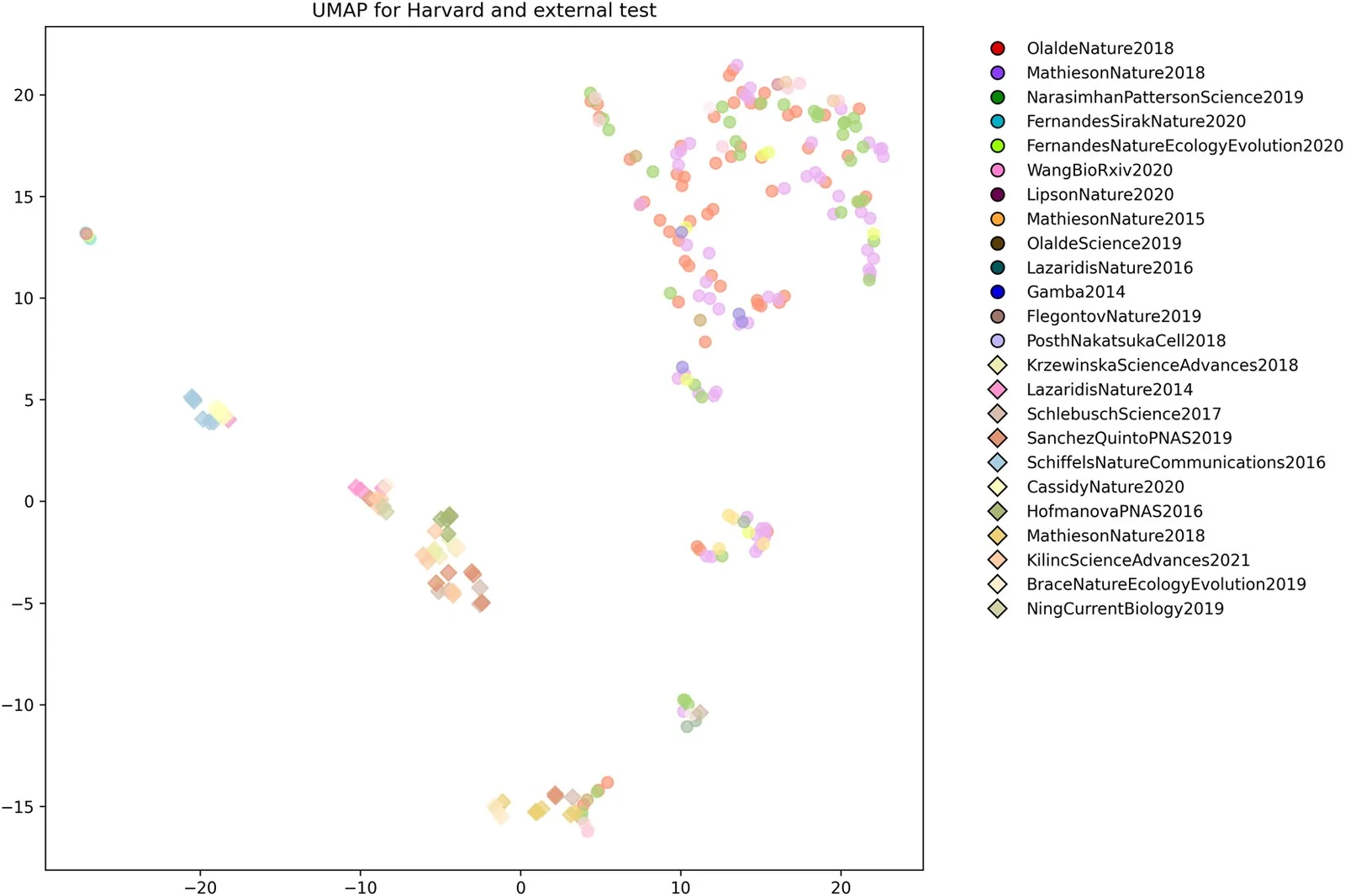
UMAP visualization of raw misincorporation profiles prior to batch correction. Samples are colored by publication source and shaped by dataset (Harvard: circles; external test dataset: diamonds). Distinct clustering by sequencing laboratory indicates the presence of substantial technical variation in damage features.

To assess the extent of technical variation in damage profiles across sequencing laboratories, we visualized the raw misincorporation features using UMAP prior to any batch correction (Fig. 2). Samples are colored by publication source and shaped by dataset (Harvard versus external test dataset). Despite representing similar archaeological contexts, samples originating from different laboratories form distinct clusters in feature space, indicating that sequencing origin introduces substantial variation in the damage profiles. In particular, samples from the external test dataset tend to form compact and well-separated clusters, suggesting systematic differences in sequencing or library preparation protocols. Notably, samples from the same publication but different datasets do not consistently co-localize, implying that technical effects may contribute substantially to the observed structure in the raw feature representation. As UMAP is a stochastic dimensionality reduction method, the exact layout of clusters may vary across runs; however, the overall separation by sequencing origin is consistently observed.

To mitigate batch effects [9], we grouped all samples according to their publication of origin, resulting in a total of 15 groups. We then partitioned the dataset into train, validation, and test subsets such that each group was assigned exclusively to one subset. This strategy ensured that no publication group was shared across splits. Notably, when samples were not grouped in this manner, the model initially appeared to perform well; however, it was learning to predict the source publication rather than the sample age.

The data from DamageProfiler were divided into [Train & Validation—Test] datasets with proportion of [0.9–0.1]. The Train & Validation subset was used further in the pipeline with the cross-validation. The mean absolute error (MAE) and *R*^2^ were used as metrics for the performance. In addition, for inference, we used BAM files not included in the Harvard dataset files to demonstrate that the algorithm generalizes well beyond the training set (no significant batch effect present). We also demonstrate the current limitations of the algorithm for the inference data.

### Pipeline

We calculated the statistics for each type of mutation transition and the transition probability for each sequence length (the typical output of the DamageProfiler file [7]).

**Figure A1:**
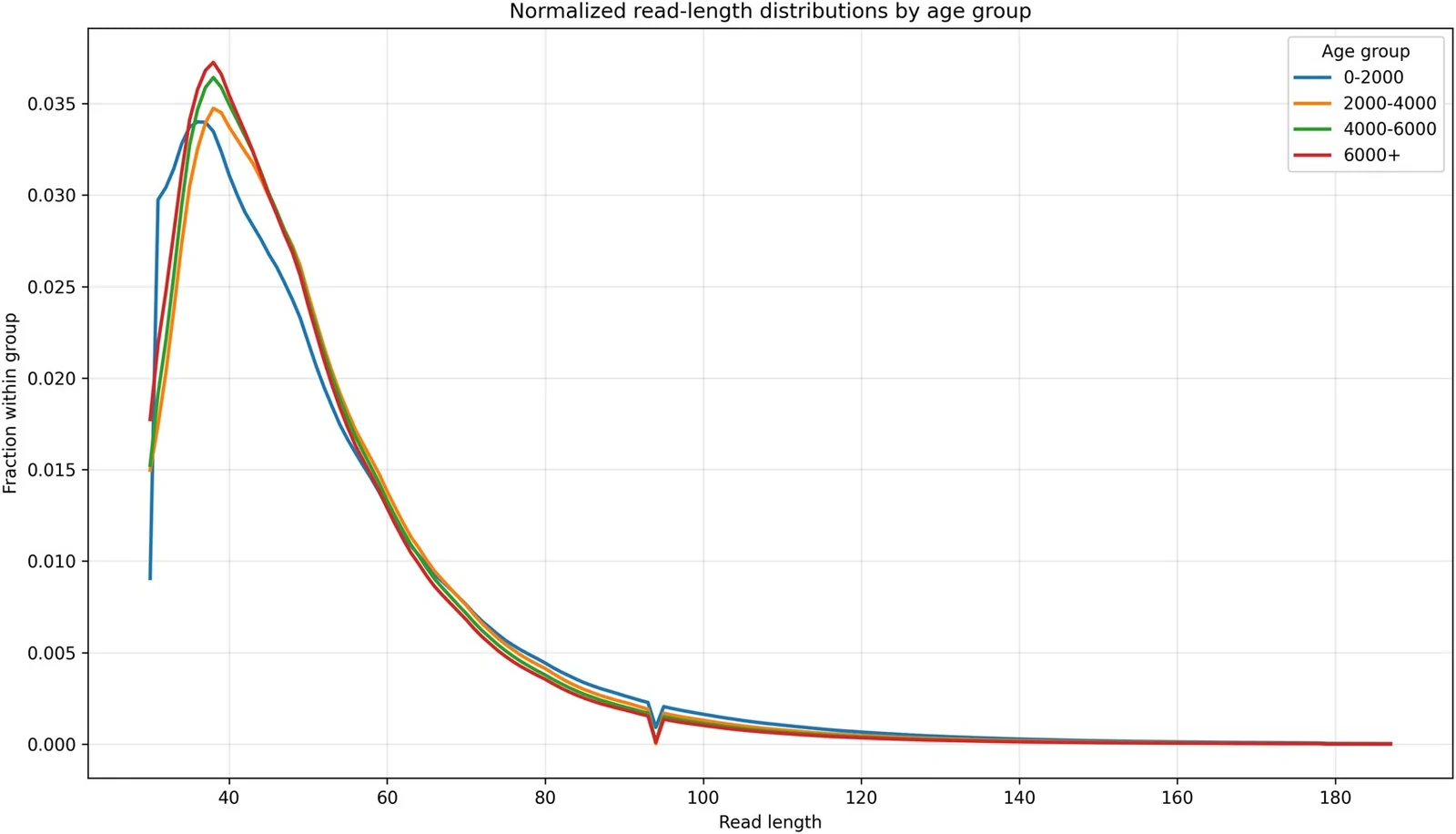
Read length distribution in Harvard dataset divided by age groups.

A total of 199 BAM files were used in this study. These 199 BAM files from the Harvard dataset were used for model training, validation, and internal testing, while the independently collected external ancient genomes were reserved exclusively for inference in order to evaluate generalization to unseen datasets. The original Harvard dataset contained 216 BAM files, but 17 were excluded due to memory constraints associated with processing individual files. Each BAM file yielded approximately 5900 features, corresponding to all combinations of mutation types and positional sequence contexts. Columns containing NaN values (approximately 400 columns) were removed. Additionally, DamageProfiler features corresponding to read positions greater than 60 (out of 100) were excluded. This truncation threshold is supported by the empirical read length distribution of the dataset (Appendix 5, Fig. A1), where the majority of sequencing reads are shorter than approximately 60 base pairs. This filtering was applied to the aggregated positional damage features computed across all reads within each BAM file, rather than to individual reads themselves. Notably, removing these positions did not substantially affect model performance.

Feature normalization was performed as follows: counts of individual nucleotides (e.g. “A,” “G”) and the “S” feature were normalized by the “TOTAL” read count; mutation transition features (e.g. “A→T,” “A→?”) were normalized by the count of the originating nucleotide (“A” in this example); and reverse transitions (e.g. “→A”) were normalized by the count of the target nucleotide. Normalization was applied row-wise to the DamageProfiler output. Finally, the “TOTAL” column was removed to prevent the model from exploiting coverage-related correlations rather than true temporal signal.

**Figure 3:**
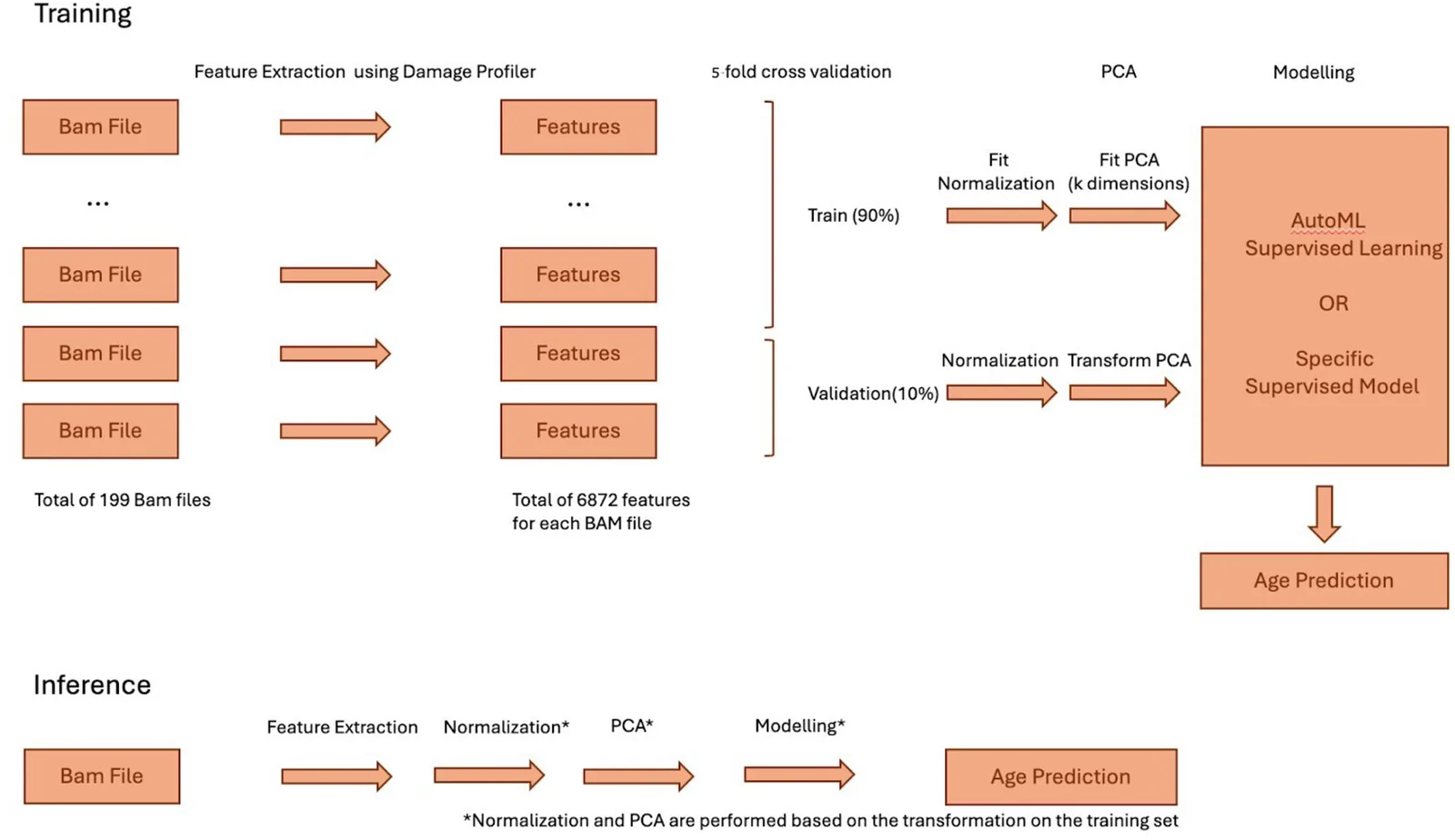
The general pipeline for the age prediction.

In our setting, each BAM file yields approximately 5900 features, while the number of available samples is limited to 199. This corresponds to a high-dimensional p≫n regime [10], in which the number of features exceeds the number of observations. In such cases, dimensionality reduction is typically required to obtain stable regression estimates. We therefore applied Principal Component Analysis (PCA) [11] prior to model training. Therefore, some kind of dimensionality reduction is necessary in order to predict the age of the sample. We used the PCA algorithm for this purpose. The use of PCA is justified in our case since the features are highly correlated. The output number of features was a hyperparameter for a pipeline (from 10 to 120). Before feeding the data into PCA, each feature was normalized to [0,1]. The general pipeline for age prediction is schematically presented in Fig. 3.

**Figure 4:**
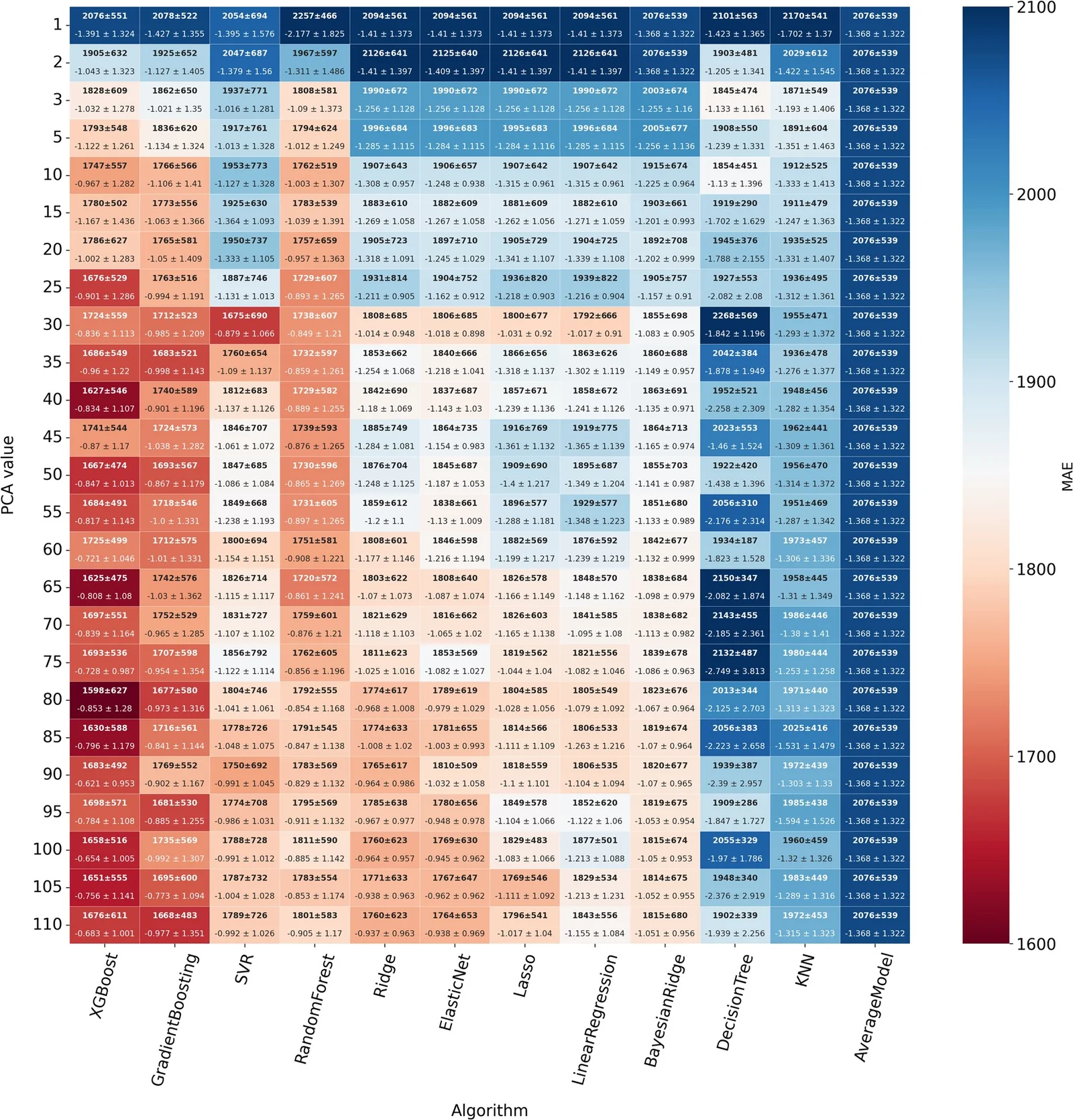
The comparison of different supervised models for predicting the age of the sample.

In this study, we evaluated multiple regression models to identify the most effective approach and corresponding hyperparameters for ancient sample age prediction. All regression models were trained using a scikit-learn Pipeline consisting of feature standardization, dimensionality reduction via PCA, and regression. The PCA transformation was fitted exclusively on the training portion of each cross-validation fold and subsequently applied to the corresponding validation fold to prevent data leakage. Model hyperparameters were optimized using GridSearchCV with grouped 5-fold cross-validation (GroupKFold), where grouping was defined by publication of origin (batch_name), ensuring that samples from the same study were not shared between training and validation folds during hyperparameter tuning or performance evaluation. The MAE was used as the optimization criterion during grid search. The number of PCA components was treated as an external hyperparameter and evaluated in the range of 10 to 120 jointly with model-specific parameters. For each evaluated configuration, model performance was estimated using cross-validated predictions obtained via cross_val_predict under the same grouped splitting scheme, and final performance metrics (MAE, RMSE, and $R^{2}$) were computed across all held-out predictions from the cross-validation procedure. The final model configuration was selected as the combination of regression algorithm and PCA dimensionality that minimized the cross-validated MAE across all grouped folds. A held-out test dataset was defined using grouped splitting by publication and was not used during either hyperparameter tuning or cross-validation. All stochastic components of the training process were controlled using a fixed random seed. We tested a range of supervised learning algorithms (summarized in Fig. 4). Models implemented via the Scikit-learn library [12] included Ridge Regression, Lasso, ElasticNet, Random Forest, Support Vector Regression (SVR), k-Nearest Neighbors (KNN), Bayesian Ridge, and Decision Trees. In addition, we incorporated the XGBoost algorithm using the XGBoost framework [13]. Model performance was compared against a baseline predictor equal to the mean age of the training set. To ensure reliable evaluation, all models were assessed using 5-fold cross-validation [14].

## Results

In Fig. 4, the results of the algorithm comparison are presented. The best-performing model was XGBoost, achieving a MAE of 1669±435 years under 5-fold cross-validation, using 50 principal components as input features. The benchmark regressor, which predicts the mean age of the training set, achieved an MAE of 2064±472 years. Although certain models and PCA configurations produced slightly lower MAEs, these differences were generally within one standard deviation. We also evaluated the selected model on the held-out external test dataset, obtaining similar results. Together, these observations indicate that the apparent performance differences are not statistically meaningful and are likely driven by noise rather than a true predictive signal. Thus, we conclude that no strong age-predictive signal is present in the training data. In several datasets, the model MAE exceeded that of the baseline mean predictor. This behavior is expected in high-dimensional settings with a weak signal and distributional shift between training and test data. In our case, grouping samples by publication introduces covariate shift in DNA damage features, causing PCA-based regression models to capture batch- or environment-specific variation rather than chronological signal. As a result, model predictions become biased on unseen datasets, whereas the baseline predictor, which depends only on the mean age of the training set, remains unaffected by such feature shifts.

**Figure 5:**
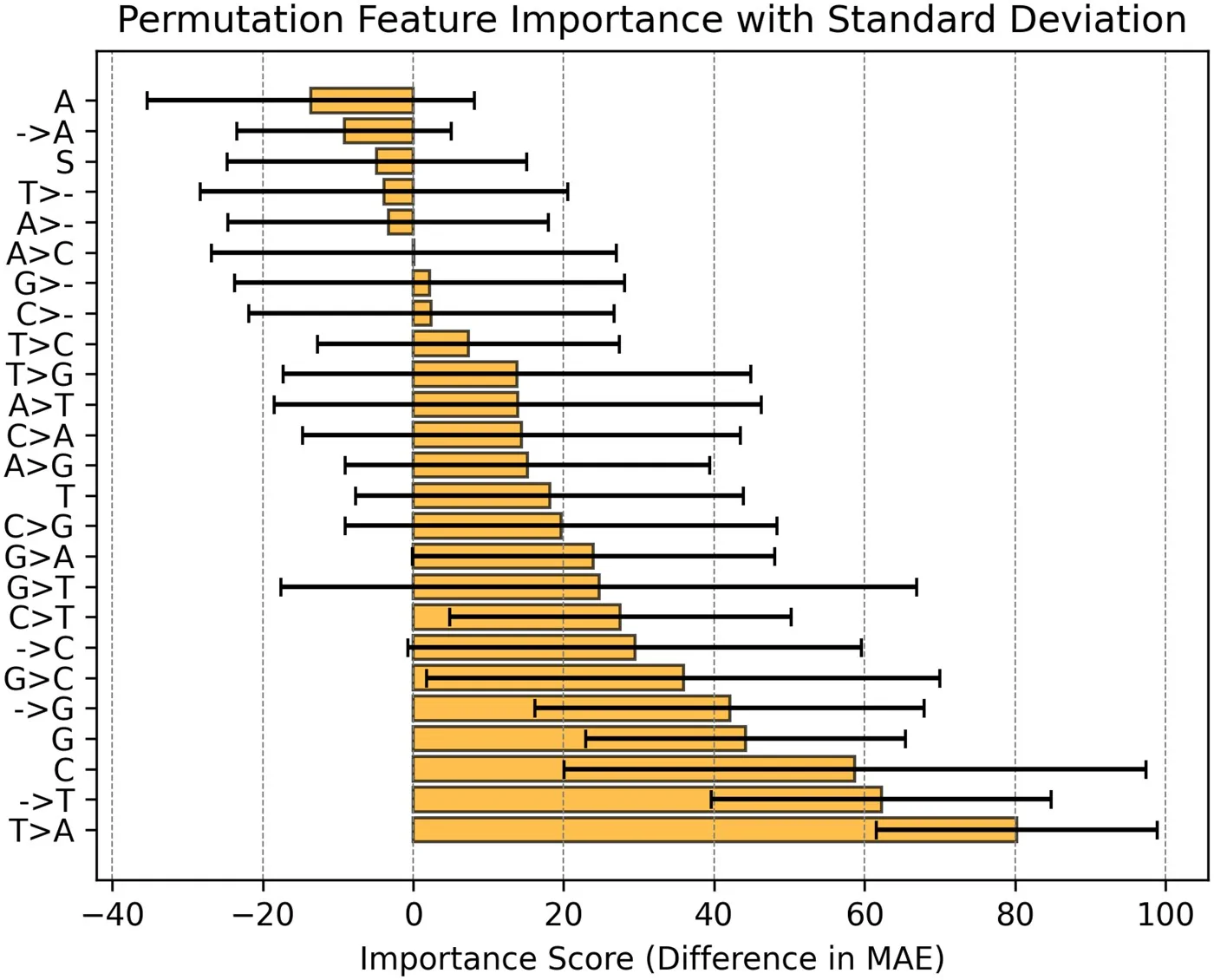
Permutation feature importance of DNA damage-derived predictors. Feature importance is quantified as the increase in MAE after randomly permuting each feature group across samples while keeping all other inputs unchanged. Positive values indicate degradation in predictive performance when the corresponding feature is disrupted. Error bars represent the standard deviation across repeated grouped cross-validation experiments.

To further investigate which damage-derived predictors were utilized by the trained model, we computed permutation feature importance, defined as the increase in MAE following random shuffling of each feature group across samples (Fig. 5). The most influential predictors included substitution types such as T→A, insertions (→T), and nucleotide content metrics including C and G counts, as well as transversions such as G→C and insertions to G (→G). Permuting these features resulted in the largest degradation in predictive performance, indicating that the trained model partially relies on them. In contrast, canonical ancient DNA damage signatures such as C→T and G→A transitions exhibited near-zero importance once grouping by publication was enforced, suggesting that their apparent predictive value in ungrouped settings may be attributable to batch-specific artifacts rather than chronological signal. Several features (e.g. A content and →A insertions) yielded slightly negative importance values, consistent with noise-dominated predictors. Importantly, most feature importance estimates were accompanied by large standard deviations overlapping zero, reflecting substantial variability across cross-validation folds and further supporting the absence of a stable age-related signal in the available damage statistics. The final performance on the internal Harvard test dataset and the external dataset is summarized in Table 1. Consistent with the cross-validation and validation results, the model fails to predict sample ages in both test settings, confirming that the age signal cannot be reliably recovered from the available features.

**Table 1:** Absolute age prediction error (years) for baseline mean predictor versus model across datasets.

Dataset	**Baseline MAE** ± **SD (years)**	**Model MAE** ± **SD (years)**
**Harvard**	1593.3 ± 967.9	1014.4 ± 899.8
**Aegean**	1633.5 ± 1111.6	2326.8 ± 1184.6
**Africa**	3660.3 ± 864.8	4356.5 ± 1202.8
**Britain**	5271.0 ± 547.7	5317.3 ± 561.9
**Cambridge**	3279.0 ± 402.5	3522.3 ± 981.3
**Caspian**	2379.0 ± 679.8	1814.5 ± 685.6
**Cassidy**	545.8 ± 358.4	787.0 ± 675.2
**Mathieson**	3887.6 ± 1291.3	3848.7 ± 1574.6
**Mittnik**	2446.0 ± 342.8	3006.0 ± 486.1
**Quinto**	527.8 ± 500.3	1352.8 ± 950.6
**Siberia**	1245.5 ± 977.6	1324.4 ± 1188.3
**Yamnaya**	2629.0 ± 70.7	3239.5 ± 1496.9

For the test dataset, we used a test subset of the Harvard dataset and additional samples outside of the Harvard dataset. We note that we did not include the test dataset in the cross-validation, so the test dataset was unseen for the algorithms. For the Harvard dataset, the results are the following. We calculated the average actual age as the average age for the test dataset. We calculated the MAE (Predicted versus Actual) as the difference between the predicted and actual age; the baseline MAE as the difference between the predicted and the average actual age.

We can see that the MAE (predicted) is more than the baseline benchmark, which indicates that the model does not do well on test data (which is part of the Harvard).

## Discussion

The supervised learning methods applied in this study did not produce a statistically significant improvement over the benchmark model. Although XGBoost achieved the lowest cross-validation MAE (1669 ± 435 years) with 50 PCA components, this value lies within one standard deviation of the benchmark regressor (2064 ± 472 years). The overlapping performance across algorithms and PCA configurations indicates that the observed differences are likely caused by random variation rather than a genuine signal.

These findings were further supported by the results on the independent test dataset, which included both the Harvard dataset test samples and external data. Model predictions showed no consistent improvement over baseline estimates derived from the mean age, suggesting limited generalization beyond the training distribution.

Interestingly, when batch effects were not addressed specifically (when data from different publications were mixed during training and evaluation), the models exhibited substantially lower prediction error and appeared to capture a strong signal (see Appendix 1). Subsequent analysis, however, demonstrated that this improvement was primarily driven by batch-specific artifacts rather than by meaningful biological patterns. In other words, the models effectively learned to distinguish the source article rather than predicting the true chronological age. This observation underscores the presence of strong confounding factors tied to data origin and highlights the importance of rigorous batch control and cross-validation across studies.

However, even after applying batch-correction techniques, the confounding signal persisted, and performance did not improve, suggesting that current batch-adjustment methods are insufficient for this task and that deeper biases may be inherent in the dataset. Overall, the present approach, which is based solely on statistical features and PCA-based dimensionality reduction, appears insufficient for capturing a robust age-related signal in the current dataset once batch effects are properly controlled. In the main analysis, positional damage features were truncated at 60 base pairs, approximately corresponding to the median read length in the dataset. This representation was chosen to ensure a consistent feature space across samples and to reduce sparsity arising from variable fragment lengths. In particular, extending the positional range introduces a large number of positions that are unobserved for shorter fragments, leading to systematic missingness and increased noise in the feature matrix. Truncating the positional range, therefore, provides a compact and stable representation that is less sensitive to coverage variability and fragment length heterogeneity.

However, this choice may attenuate damage signals originating from the distal ends of reads, especially in UDG-treated libraries where deamination patterns are localized near fragment termini. To evaluate this effect, we conducted additional experiments using the full positional range of DamageProfiler outputs (Appendix 2). These results show that preserving the full positional information does not improve predictive performance, suggesting that the absence of a strong age-related signal is not driven by the positional truncation but rather reflects the intrinsic limitations of the available damage features.

Nonetheless, this outcome does not preclude the possibility of success with alternative strategies. Future work may benefit from incorporating domain-specific biological features, exploring non-statistical representations, or integrating complementary data sources. Additionally, expanding the dataset size could help distinguish the true signal from noise and improve model robustness.

An additional source of uncertainty arises from the chronological labels themselves. The sample ages used as ground truth in this study are derived from heterogeneous sources, primarily radiocarbon dating and archaeological context reported in the original publications. These estimates are subject to methodological uncertainties, including calibration errors, laboratory-specific differences, and contextual interpretation. Moreover, differences in dating protocols across studies may limit the comparability of age estimates between datasets. As a result, the target variable in our regression setting may contain non-negligible noise, which could further obscure any weak relationship between DNA damage patterns and chronological age.

In addition to genetic features, we explored the use of meteorological data as auxiliary input. Previous work [4] has demonstrated that environmental conditions can influence DNA degradation rates. To account for this, we incorporated average precipitation and temperature values into our pipeline. However, under grouped (batch-out) evaluation, meteorological features did not provide a predictive signal and, in some cases, performed worse than the baseline predictor. A detailed description of the experimental setup and corresponding results is provided in Appendix 3.

Our method produced largely negative results across multiple batches. From a biophysical perspective, one likely explanation for the inability to predict the age of the samples lies in the dynamic nature of climate: DNA degradation is driven not by a steady process, but by daily and seasonal temperature fluctuations, humidity variations, and long-term environmental shifts. These dynamic conditions mask a clear relationship between molecular damage and chronological time.

To assess whether chronological age is associated with canonical ancient DNA damage patterns, we performed a correlation analysis using cytosine deamination statistics from the DamageProfiler outputs. Two commonly used damage indicators were evaluated: (i) the frequency of C→T substitutions at the first nucleotide position of sequencing reads (5′ position 1), and (ii) the mean C→T substitution frequency across the first ten bases from the 5′ read end. Across the analyzed dataset (n=188 samples), the correlation between sample age and the C→T frequency at position 1 was weak (Pearson r=0.1598, p=0.0285; Spearman ρ=0.2649, $p=2.39\times 10^{-4}$). Using the mean C→T frequency across the first ten bases produced similarly small associations (Pearson r=0.1804, p=0.0132; Spearman ρ=0.3045, $p=2.15\times 10^{-5}$). These effect sizes are small: e.g. r=0.1598 corresponds to $r^{2}\approx 0.025$, indicating that chronological age explains only about 2.5% of the variance in the damage metric. Despite these statistically significant but weak correlations, supervised learning models failed to extract a reliable predictive signal from the damage profiles. An additional experiment restricting the input to canonical damage patterns (C→T at the 5${\;}^{\prime }$ end and G→A at the 3${\;}^{\prime }$ end) likewise did not improve predictive performance (Appendix 7). In fact, results were slightly worse than in the full-feature setting and remained comparable to the baseline predictor, further supporting the conclusion that the absence of predictive signal is not due to the inclusion of non-informative mutation types but reflects intrinsic limitations of the damage-derived features. This conclusion remains unchanged when restricting the analysis to non-UDG-treated samples, where deamination signals are preserved (Appendix 2). None of the evaluated models consistently outperformed a baseline predictor based on the mean sample age, indicating that the magnitude of the observed association is insufficient for accurate age prediction once environmental and technical variability are taken into account. Nevertheless, this does not imply that the task is impossible. With larger, cleaner datasets and improved control over environmental and technical variability (proper batch-effect), it may become feasible to extract reliable temporal signals from DNA damage profiles in future research.

## Conclusion

Our study evaluated the potential of using machine learning to estimate the chronological age of ancient DNA samples based on their molecular damage profiles. Once batch effects were controlled, the apparent predictive signal largely disappeared, indicating that experimental and environmental factors dominate the variation in DNA damage patterns. None of the tested algorithms consistently outperformed a simple mean-based baseline.

These findings suggest that damage profiles alone are insufficient for reliable age estimation. However, they also highlight important directions for future research. Incorporating additional biological and contextual information, such as preservation environment, biochemical parameters, and physical models of DNA decay, could improve predictive power. Expanding dataset size and diversity may further help distinguish genuine chronological signals from background noise. Thus, while our current approach did not achieve accurate molecular dating, it provides a benchmark and methodological insight for subsequent developments in the field.

All code used in this work is publicly available at the GitHub repository.

## Figures and Tables

**Figure A2:**
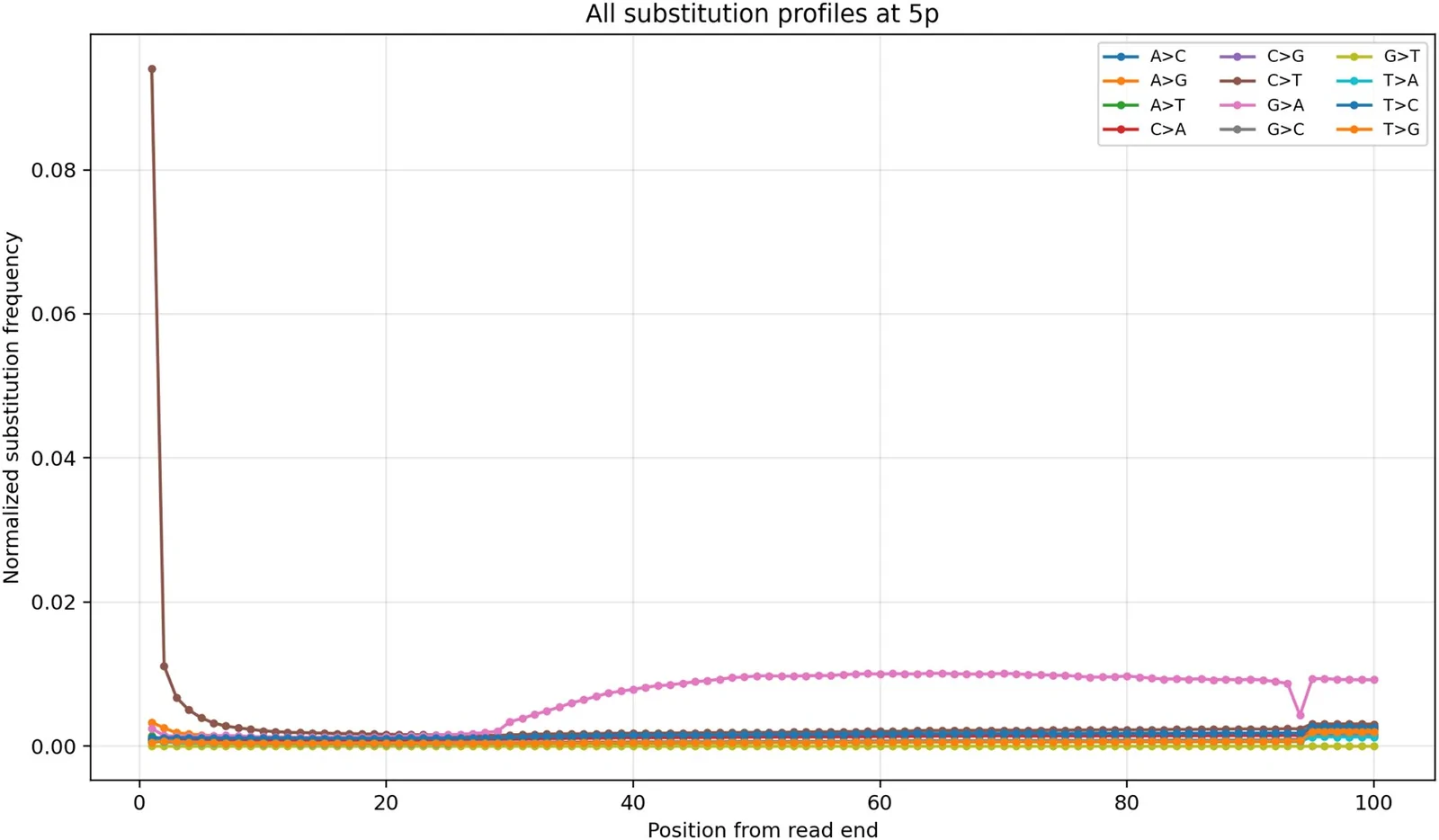
Damage profile from the 5′-end of sequencing reads.

**Figure A3:**
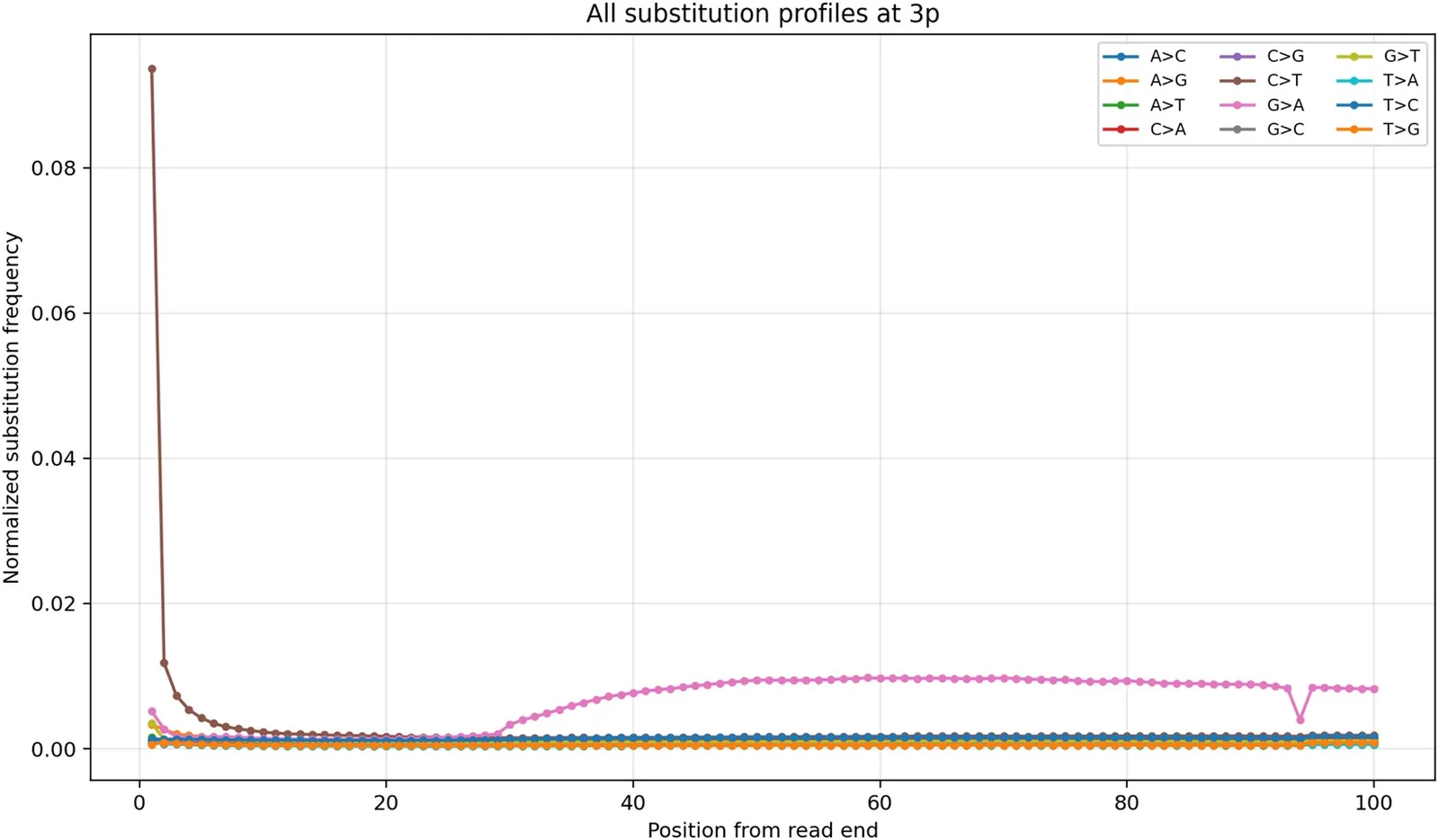
Damage profile from the 3′-end of sequencing reads.

**Figure A4:**
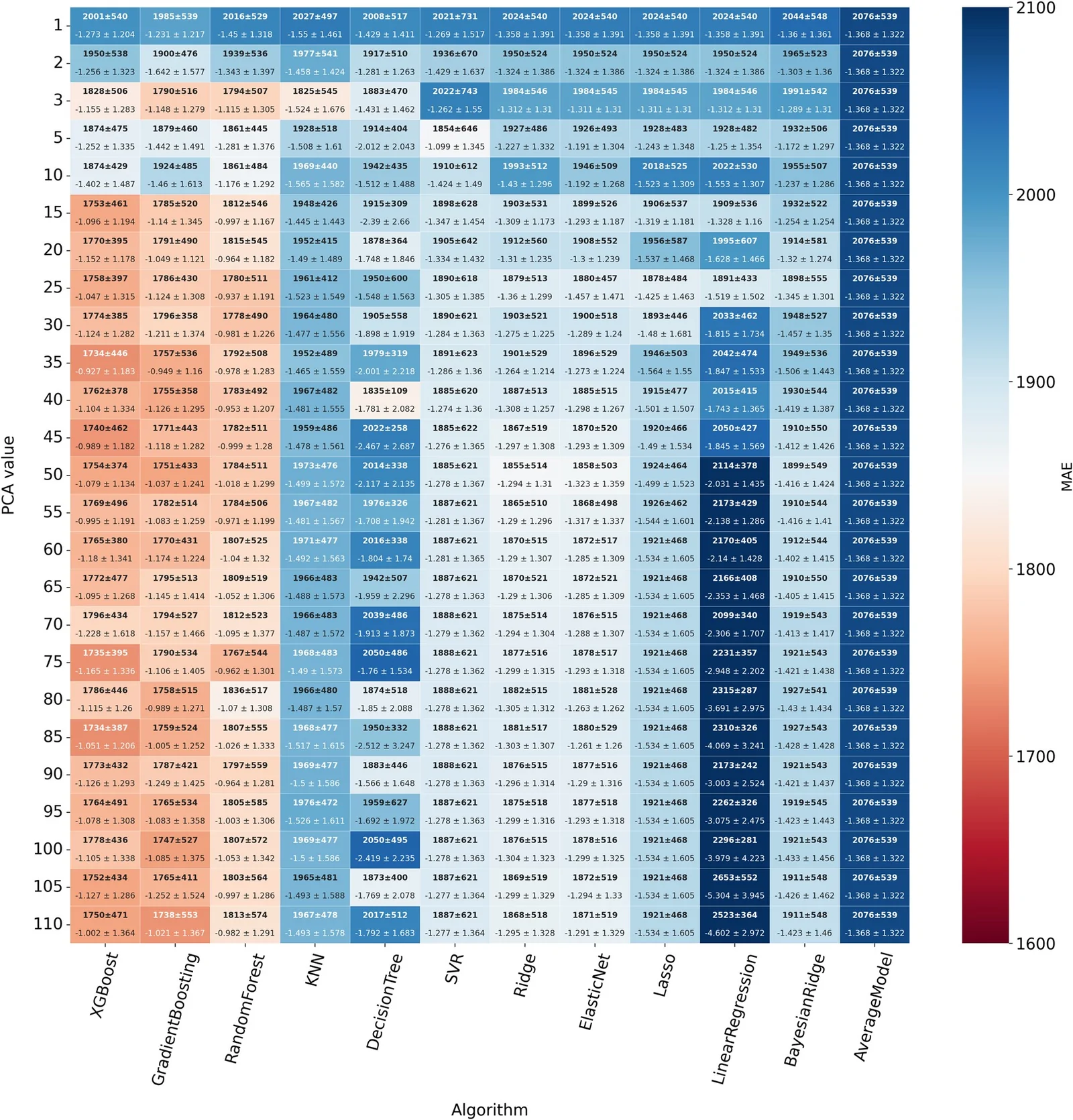
Performance of regression models using canonical damage features only. Heatmap of MAE across different regression algorithms and PCA dimensionalities, using only canonical post-mortem damage patterns (C→T at the 5′-end and G→a at the 3′-end) as input features. Each cell reports the mean MAE (in years) ± standard deviation obtained under grouped cross-validation .

## Data Availability

The ancient DNA datasets analyzed in this study were obtained from publicly available sources, including the Allen Ancient Genome Diversity Project (AAGDP), the European Nucleotide Archive (ENA), and the NCBI Sequence Read Archive (SRA). Accession numbers for all samples used in this study are provided in Appendix 4. The processed datasets generated during this study, including DamageProfiler-derived feature tables, sample metadata, chronological age labels, batch assignments, and machine-learning input matrices, are publicly available. All source code required to reproduce the analyses, figures, tables, and machine-learning experiments reported in this manuscript is available at: GitHub repository: https://github.com/maksimkazanskii/DNA_age_prediction Zenodo archive: https://doi.org/10.5281/zenodo.20458891 The Zenodo archive provides a permanent, citable version of the software and processed data used in this study. Reproduction of the analyses described in this manuscript does not require access to the original BAM files, as all processed DamageProfiler outputs and derived feature matrices used for model development and evaluation are included in the archived dataset.

## Appendix 1. Batch effect evaluation

The dataset consists of samples originating from 14 distinct batches (corresponding to different publications) of the Harvard dataset, each of which may introduce systematic batch-specific effects. To investigate the influence of batch structure on model performance, we conducted two experimental setups. All experiments were performed using only the Harvard dataset (199 samples). In the leave-one-batch-out (LOBO) validation setting, batch structure is explicitly taken into account: for each of the 14 batches, one batch is designated as the test dataset while the remaining 13 batches are used for training. Within the training data, hyperparameter tuning is performed using grouped cross-validation, where splits are constructed such that samples from the same batch are not shared between training and validation folds. After selecting the optimal hyperparameters, the model is retrained on the full training set and evaluated on the held-out batch. This procedure is repeated for all batches, and the final performance is reported as the average across all test batches. The detailed per-batch results for this setup are presented in Appendix Table A1. In the second setup, batch structure is not considered: all samples are pooled together, and a standard random 5-fold cross-validation is performed, allowing samples from the same batch to appear in both training and validation splits. Hyperparameters are selected within this procedure, and performance is reported as the mean across folds. The results for this batch-neglected setting are summarized in Appendix Table A2.

The comparison between these two evaluation strategies provides an empirical assessment of the effect of batch structure on performance estimates, as reflected by the difference between the LOBO results and the random cross-validation results reported in Appendix Tables A1 and A2. The results demonstrate that, under the LOBO protocol, model performance remains comparable to the baseline, indicating limited generalization across batches. However, when the batch structure is ignored (Appendix Table A2), performance improves substantially. This behavior suggests that the model is primarily capturing batch-specific signals rather than the underlying target variable.

## Appendix 2. Effect of UDG treatment on age prediction performance

Uracil-DNA glycosylase (UDG) treatment is commonly applied in ancient DNA workflows to remove deaminated cytosines and reduce sequencing errors; however, cytosine deamination (observed as C→T transitions) represents one of the primary molecular signatures of post-mortem DNA damage and is often considered a potential proxy for sample age. To assess whether removal of this signal affects predictive performance, we conducted an additional experiment restricted to non-UDG-treated samples, in which deamination patterns are preserved. The experimental setup follows the same pipeline as described in the main text, including feature extraction from DamageProfiler outputs, normalization, dimensionality reduction via PCA, and grouped cross-validation (GroupKFold); however, in this case only a single regression model (XGBoost) was evaluated instead of the full set of algorithms. The filtered dataset consisted of n=29 samples grouped into seven batches, and the number of PCA components was varied to evaluate its effect on performance. The results are summarized in Appendix Table A3. The best-performing configuration (PCA = 20) achieved a MAE of 2802.15±1006.10 years, while the baseline predictor (mean age of the training set) achieved 2420.39±721.72 years. Across all tested configurations, model performance remained comparable to or worse than the baseline. These results indicate that preserving cytosine deamination signals alone is insufficient to recover a reliable relationship between DNA damage features and chronological age, suggesting that environmental variability and data limitations dominate the observed patterns even in the absence of UDG treatment; however, it should be noted that the relatively small sample size of the non-UDG subset limits statistical power, and therefore these results should be interpreted with caution.

Extended positional feature experiment: To evaluate whether truncation of positional features affects predictive performance, we conducted an additional experiment in which the positional cutoff was removed and the full positional range of DamageProfiler outputs was used. The experimental setup remained identical to that described above, including normalization, PCA-based dimensionality reduction, and grouped cross-validation (GroupKFold).

The best-performing configuration achieved a MAE of 2802.15 years. The PCA-wise performance is summarized in Appendix Table A4. Consistent with the results obtained under positional truncation, no configuration resulted in a meaningful improvement over the baseline predictor. These findings indicate that preserving distal read-end damage signals does not improve age prediction performance in the non-UDG subset.

## Appendix 3. Meteorological feature experiments

To assess the potential contribution of environmental conditions to age prediction, we conducted a set of controlled experiments using meteorological variables associated with each sample. Specifically, we incorporated two features: average annual temperature (tavg) and precipitation (prcp). To obtain these features, we utilized an automated Python pipeline. Sample origin names were converted into geographic coordinates using the geopy library, which were then used to query the meteostat library to identify the nearest active meteorological station. For each site, we extracted historical 20th-century records and calculated a temporal average to establish a stable climatic baseline. The purpose of this analysis was to evaluate whether environmental variables alone carry predictive signal for sample age, and to determine whether they improve performance when combined with genetic damage features.

We considered three experimental configurations:

- Baseline (mean predictor): A constant model predicting the mean age of the training data within each fold.
- Meteo model: A regression model trained exclusively on meteorological features (tavg, prcp).
- Full model: A model combining genetic damage features with meteorological variables.

All models were evaluated using grouped cross-validation (GroupKFold), where grouping was defined by publication (batch_name). This ensures that samples from the same study are not shared between training and validation folds, thereby preventing information leakage and providing a realistic estimate of generalization to unseen datasets.

For each fold, models were trained on all but one batch and evaluated on the held-out batch. Performance metrics were computed independently for each fold, including MAE, root mean squared error (RMSE), and coefficient of determination ($R^{2}$). Final results are reported as the mean and standard deviation across folds. The meteo-only model was implemented using XGBoost without dimensionality reduction, as the feature space consists of only two variables. In contrast, the full model retained the same pipeline as described in the main text, including feature standardization, PCA applied to genetic features, and gradient boosting regression. The results of these experiments are summarized in Appendix Table A5. The meteo-only model performs worse than the baseline predictor across all metrics, indicating that average temperature and precipitation alone do not provide a meaningful predictive signal for sample age under grouped evaluation. The full model shows only marginal improvement over the baseline and remains within 1 SD, suggesting that the inclusion of meteorological features does not significantly enhance predictive performance when combined with genetic damage features as described in the main paper methods.

These findings further support the conclusion that environmental variability contributes primarily as noise rather than as a stable predictor of chronological age in the current dataset. Moreover, they highlight that apparent correlations between environmental factors and DNA damage do not translate into a robust predictive signal once batch effects and dataset stratification are properly controlled.

## Appendix 4 Ancient DNA samples used in this study

### Column descriptions

Batch: Study or dataset identifier indicating the publication or source group to which the sample belongs.

Age1950: Estimated age of the individual in years before 1950 (the standard archaeological reference year).

Run accession: Sequence run identifier from public archives (e.g. ENA/SRA) corresponding to the specific BAM file.

Study accession: Project-level accession identifier for the study in which the sample was generated.

Reference: Citation for the original publication reporting and describing the ancient DNA data.

A complete list of ancient DNA samples included in the study is provided in Appendix Table A6. For each sample, we report the dataset of origin, estimated age before 1950, sequencing run accession, study accession, and the corresponding original publication.

## Appendix 5 Distribution of read length in dataset

[Please place Fig. A1 and Table A7].

## Appendix 6 Damage frequencies from 5′ and 3′ read ends

Figures A2 and A3 illustrate the average nucleotide misincorporation patterns observed at the 5′${\;}^{\prime }$ and 3′ read termini. Although the expected ancient DNA damage signatures are clearly present, substantial variability across samples obscures any robust relationship between damage intensity and chronological age.

## Appendix 7 Canonical damage signal representation

To address the concern that including all transitions and indels may dilute the biologically relevant damage signal, we performed an additional experiment in which the input was restricted to canonical post-mortem damage patterns only—specifically C→T transitions at the 5′-end and G→A transition at the 3${\;}^{\prime }$ end. These features were retained in a position-aware manner, analogous to the standard *5pCtoT_freq* representation, by preserving their profiles as a function of distance from read ends. Importantly, this experiment was otherwise identical to the main pipeline (same normalization, PCA-based dimensionality reduction, grouped cross-validation, and model evaluation), ensuring a controlled comparison in which only the feature representation was modified. The results are summarized in Fig. A4. Across all tested algorithms and PCA configurations, predictive performance was slightly worse than in the full-feature setting and remained comparable to the baseline model. No configuration demonstrated a consistent reduction in MAE, and performance differences were within one standard deviation. These findings indicate that restricting the model to canonical damage signals does not improve predictive performance and does not reveal a meaningful relationship between DNA damage and chronological age under the current data conditions.

**Table A1:** LOBO results across 14 batches. Bold values correspond to the Average and SD rows, which summarize the mean and standard deviation of performance across all evaluated batches.

Batch	XGB CV MAE	XGB Test MAE	Baseline CV MAE	Baseline Test MAE
**OlaldeNature2018**	2166.07	1329.89	2075.29	1336.82
**MathiesonNature2018**	1441.50	2624.54	1393.06	2855.90
**NarasimhanPattersonScience2019**	2024.21	1916.39	1923.12	1505.60
**FernandesSirakNature2020**	1780.65	4724.55	1707.82	4590.97
**FernandesNatureEcologyEvolution2020**	1919.34	1559.58	1770.73	1370.43
**WangBioRxiv2020**	1666.69	2485.52	1728.36	2422.88
**LipsonNature2020**	1710.15	3030.23	1748.03	2408.00
**MathiesonNature2015**	1860.37	3113.39	1720.27	2913.96
**OlaldeScience2019**	1673.15	2435.18	1749.09	2210.14
**LazaridisNature2016**	1678.52	2073.03	1727.57	2954.23
**Gamba2014**	1837.34	800.93	1754.47	1622.19
**PosthNakatsukaCell2018_A**	1703.90	633.80	1774.34	410.76
**FlegontovNature2019**	1693.58	3641.89	1731.42	4558.03
**PosthNakatsukaCell2018_B**	1700.90	4475.11	1712.20	5645.00
**Average**	**1775.45**	**2488.86**	**1751.13**	**2628.92**
**SD**	**175.61**	**1235.01**	**115.86**	**1465.89**

**Table A2:** Random 5-fold cross-validation results (batch-neglected setting) and performance on a held-out 15% test dataset. The bold formatting is used to highlight the XGBoost model name..

Model	**CV MAE (mean** ± **std)**	Test MAE
**XGBoost**	**1290.23** ± **200.26**	**1328.29**
**Baseline**	1757.39 ± 162.61	1510.42

**Table A3:** Model performance (MAE in years) on non-UDG-treated samples for different PCA dimensionalities. Bold values indicate the best (lowest) mean absolute error (MAE) obtained across all tested PCA dimensionalities.

PCA Components	**XGBoost MAE** ± **SD (years)**
**1**	3123.14 ± 721.11
**2**	3173.05 ± 1039.70
**3**	3383.60 ± 1022.73
**5**	3274.92 ± 1171.66
**10**	3083.55 ± 1098.27
**15**	2968.05 ± 1003.01
**20**	**2802.15** ± **1006.10**

**Table A4:** Model performance (MAE in years) on non-UDG-treated samples using the full positional range (no truncation). Bold values indicate the best (lowest) mean absolute error (MAE) obtained across all tested PCA dimensionalities..

PCA Components	**XGBoost MAE** ± **SD (years)**
**1**	3060.88 ± 60.30
**2**	3140.56 ± 85.87
**3**	3414.31 ± 68.34
**5**	3359.88 ± 143.03
**10**	3143.47 ± 58.41
**15**	3036.33 ± 88.95
**20**	**2921.90** ± **73.53**

**Table A5:** Batch-out cross-validation performance (mean ± SD).

Model	MAE	RMSE	$R^{2}$
**Baseline (Mean Predictor)**	2147.458±603.506	2420.070±647.621	−1.292±1.268
**Meteo (tavg, prcp)**	2275.797±414.703	2774.431±381.868	−2.442±1.975
**Full (Genetic + Meteo)**	1995.431±616.356	2487.140±765.941	−1.212±1.002

**Table A6:** Ancient DNA samples used in this study.

Batch	Age1950	Run Accession	Study Accession	References
**Aegean**	7450	ERR1144001	PRJEB11848	[15]
**Aegean**	5550	ERR1144003	PRJEB11848	[15]
**Aegean**	7500	ERR1144004	PRJEB11848	[15]
**Aegean**	7250	ERR1144005	PRJEB11848	[15]
**Aegean**	5550	ERR1352893	PRJEB11848	[15]
**Africa**	1900	ERR2126368	PRJEB22660	[16]
**Africa**	2100	ERR2126372	PRJEB22660	[16]
**Africa**	500	ERR2126373	PRJEB22660	[16]
**Africa**	400	ERR2126374	PRJEB22660	[16]
**Africa**	400	ERR2126375	PRJEB22660	[16]
**Africa**	2050	ERR2126377	PRJEB22660	[16]
**Africa**	350	ERR2126379	PRJEB22660	[16]
**Britain**	10450	ERR3158025	PRJEB31249	[17]
**Britain**	9250	ERR3158027	PRJEB31249	[17]
**Britain**	10250	ERR3158029	PRJEB31249	[17]
**Britain**	9350	ERR3158042	PRJEB31249	[17]
**Britain**	10550	ERR3158103	PRJEB31249	[17]
**Cambridge**	2050	ERR405812	PRJEB4604	[18]
**Cambridge**	1300	ERR405828	PRJEB4604	[18]
**Cambridge**	1300	ERR405829	PRJEB4604	[18]
**Cambridge**	2000	ERR405830	PRJEB4604	[18]
**Cambridge**	1250	ERR563640	PRJEB4604	[18]
**Cambridge**	2150	ERR885248	PRJEB6915	[18]
**Cambridge**	1550	ERR914274	PRJEB6915	[18]
**Caspian**	1900	ERR2684379	PRJEB27628	[19]
**Caspian**	2800	ERR2684383	PRJEB27628	[19]
**Caspian**	3400	ERR2684389	PRJEB27628	[19]
**Caspian**	3500	ERR2684396	PRJEB27628	[19]
**Caspian**	2500	ERR2684399	PRJEB27628	[19]
**Caspian**	2250	ERR2684404	PRJEB27628	[19]
**Caspian**	1850	ERR2684412	PRJEB27628	[19]
**Caspian**	1800	ERR2684413	PRJEB27628	[19]
**Cassidy**	4800	ERR4057227	PRJEB36854	[20]
**Cassidy**	4150	ERR4057266	PRJEB36854	[20]
**Cassidy**	4300	ERR4057298	PRJEB36854	[20]
**Cassidy**	4050	ERR4057311	PRJEB36854	[20]
**Cassidy**	4600	ERR4057326	PRJEB36854	[20]
**Cassidy**	5850	ERR4057384	PRJEB36854	[20]
**Cassidy**	4600	ERR4057386	PRJEB36854	[20]
**Cassidy**	4400	ERR4057450	PRJEB36854	[20]
**Cassidy**	5250	ERR4057670	PRJEB36854	[20]
**Mathieson**	9200	ERR2216514	PRJEB22652	[21]
**Mathieson**	9200	ERR2216557	PRJEB22652	[21]
**Mathieson**	10050	ERR2216565	PRJEB22652	[21]
**Mathieson**	6350	ERR2216629	PRJEB22652	[21]
**Mathieson**	8700	ERR2216743	PRJEB22652	[21]
**Mathieson**	8450	ERR2216783	PRJEB22652	[21]
**Mathieson**	8100	ERR2216797	PRJEB22652	[21]
**Mathieson**	8000	ERR2216835	PRJEB22652	[21]
**Mathieson**	10850	ERR2216839	PRJEB22652	[21]
**Mittnik**	7200	ERR577490	PRJEB6272	[22]
**Mittnik**	6900	ERR577491	PRJEB6272	[22]
**Mittnik**	7550	ERR577492	PRJEB6272	[22]
**Mittnik**	7650	ERR577498	PRJEB6272	[22]
**Quinto**	5400	ERR3256979	PRJEB31045	[23]
**Quinto**	5100	ERR3258438	PRJEB31045	[23]
**Quinto**	4700	ERR3258439	PRJEB31045	[23]
**Quinto**	5450	ERR3258442	PRJEB31045	[23]
**Quinto**	6800	ERR3258445	PRJEB31045	[23]
**Quinto**	5200	ERR3258446	PRJEB31045	[23]
**Quinto**	5450	ERR3258447	PRJEB31045	[23]
**Quinto**	5650	ERR3258449	PRJEB31045	[23]
**Siberia**	1400	ERR4337441	PRJEB39378	[24]
**Siberia**	6400	ERR4351611	PRJEB39378	[24]
**Siberia**	2500	ERR4351620	PRJEB39378	[24]
**Siberia**	6500	ERR4352059	PRJEB39378	[24]
**Siberia**	7450	ERR4352061	PRJEB39378	[24]
**Siberia**	7650	ERR4352064	PRJEB39378	[24]
**Siberia**	3000	ERR4354264	PRJEB39378	[24]
**Siberia**	3800	ERR4354265	PRJEB39378	[24]
**Yamnaya**	2200	ERR3327975	PRJEB32336	[25]
**Yamnaya**	2250	ERR3327978	PRJEB32336	[25]
**Yamnaya**	2300	ERR6504206	PRJEB32336	[25]
**Yamnaya**	2200	ERR6504212	PRJEB32336	[25]

**Table A7:** Summary statistics of read length by age group.

Age group	*N* samples	Total reads	Mean length	SD	Median length
0–2000	8	3893784	49.499	20.229	46.000
2000–4000	45	19672739	49.452	18.072	46.324
4000–6000	70	29870284	48.227	17.265	45.575
6000+	65	26919322	47.229	16.852	44.966
